# Prevention and Post-extubation Screening of Perioperative Traumatic Dental Injury in Pediatric Anesthesia

**DOI:** 10.7759/cureus.95628

**Published:** 2025-10-28

**Authors:** Iqra Ahmad, Christof N Gault, Zeyad Kamar, Sharif Mohamed

**Affiliations:** 1 Department of Anesthesiology, University of Texas Medical Branch, Galveston, USA; 2 Department of Neuroscience, The University of Texas at Austin, Austin, USA; 3 Department of Medicine, Ankara Medipol University, Ankara, TUR

**Keywords:** adenotonsillectomy, airway management, dental injury, dental screening, extubation assessment, patient safety, pediatric anesthesia, pediatric surgery, primary teeth, tooth avulsion

## Abstract

Adenotonsillectomy is a routine pediatric procedure often requiring general anesthesia and endotracheal intubation. While generally safe, traumatic dental injuries (TDIs) represent a significant and often under-recognized complication, particularly in children with mobile primary teeth and anatomical risk factors. Although preoperative dental evaluations are commonly performed, current anesthetic protocols rarely include post-extubation dental screening. We present the case of a five-year-old male who underwent an uncomplicated adenotonsillectomy and was later found to have an avulsed and ingested lower central incisor, identified postoperatively by a caregiver. This case underscores the need for targeted oral examinations immediately after extubation, with attention to the anterior teeth and signs of fresh gingival trauma. We highlight the importance of both pre- and post-intubation dental assessments in pediatric patients and advocate for the adoption of structured protocols that improve early recognition of dental injuries, reduce medico-legal risk, and enhance patient safety.

## Introduction

Adenotonsillectomy is one of the most commonly performed medical procedures in the United States, with over 500000 cases annually in children under the age of 15 [1]. It involves the complete removal of the tonsils and their capsule, typically under general anesthesia with endotracheal intubation [2]. While the procedure is generally safe, with a low incidence of serious complications, adverse events can occur at any stage of care. These include postoperative pain, nausea, vomiting, hemorrhage, respiratory decompensation, and in rare cases, velopharyngeal incompetence and subglottic stenosis [3]. An additional, often overlooked complication is traumatic dental injury (TDI), which can occur during intubation and extubation, particularly in the pediatric population.

TDIs are among the most frequent anesthesia-related adverse events and represent a significant source of malpractice claims against anesthesiologists [4]. In children receiving general anesthesia, the incidence of dental trauma has been reported as high as 16% [5]. Pediatric patients are especially vulnerable due to their dentition’s anatomical and developmental characteristics, immature roots, increased overjet, anterior crowding, and ectopic eruptions, particularly between the ages of six and eight [4]. These conditions make primary teeth more mobile and more susceptible to displacement or avulsion when mechanical pressure is applied during airway manipulation.

The most common mechanism of injury is direct trauma to the anterior maxillary teeth, especially the central incisors, during laryngoscopy. Although proper laryngoscopic technique directs the Macintosh blade forward and upward to avoid dental contact, inadvertent backward tilting or excessive leverage on the upper incisors can still occur, particularly among less experienced providers or in difficult airway situations. Such forces may result in dental trauma, including fracture, subluxation, or avulsion, especially in pediatric patients with mobile primary teeth or pre-existing dental instability [4].

Several techniques have been developed to reduce the incidence of dental trauma. The paraglossal approach using a straight Miller blade inserted from the right corner of the mouth allows for glottic visualization while avoiding contact with the incisors [6]. Videolaryngoscopy has been shown to significantly reduce the rate of dental complications compared to direct laryngoscopy [7]. In high-risk patients, such as those with loose teeth, dental restorations, or difficult airways, using elastic mouthguards provides an additional layer of protection [8].

Prevention begins with a structured preoperative dental assessment, including a visual and tactile inspection for loose or damaged teeth, particularly in children with primary dentition [4]. Identifying dental instability preoperatively allows the anesthesia team to plan accordingly. However, detection should not end there. Despite growing awareness of pre-intubation risk factors, dental screening remains inconsistently performed post-extubation. Several anesthesia safety guidelines, including recommendations from the American Society of Anesthesiologists (ASA) and pediatric airway management reviews, suggest performing a targeted postoperative oral inspection to detect dental injury. The inspection should focus on the upper and lower central incisors and look for signs such as fresh gum exposure or missing teeth, particularly in patients with difficult airways or known dental mobility [9]. 

This article has been submitted as a meeting abstract at the 2025 ASA Annual Scientific Meeting on October 11, 2025.

## Case presentation

A five-year-old male weighing 16.1 kg presented for a tonsillectomy and adenoidectomy due to chronic tonsillar hypertrophy and associated obstructive sleep apnea. His past medical history includes left congenital glaucoma, for which he is being treated by a pediatric ophthalmologist. He has no prior surgical history, and the preoperative airway assessment was within normal limits for his age, with dentition intact and no loose teeth noted on initial examination.

The anesthetic plan included general anesthesia with inhalation induction using sevoflurane in oxygen, followed by intravenous access and muscle relaxation to facilitate laryngoscopy and intubation. The initial intubation attempt was performed by the CRNA using a 5.0 mm cuffed endotracheal tube (ETT) with an intubating stylet, but the ETT could not be advanced due to resistance at the subglottic level, raising concern for subglottic narrowing. The attending anesthesiologist then performed a second attempt and successfully advanced the same 5.0 mm cuffed ETT using direct laryngoscopy.

**Figure 1:**
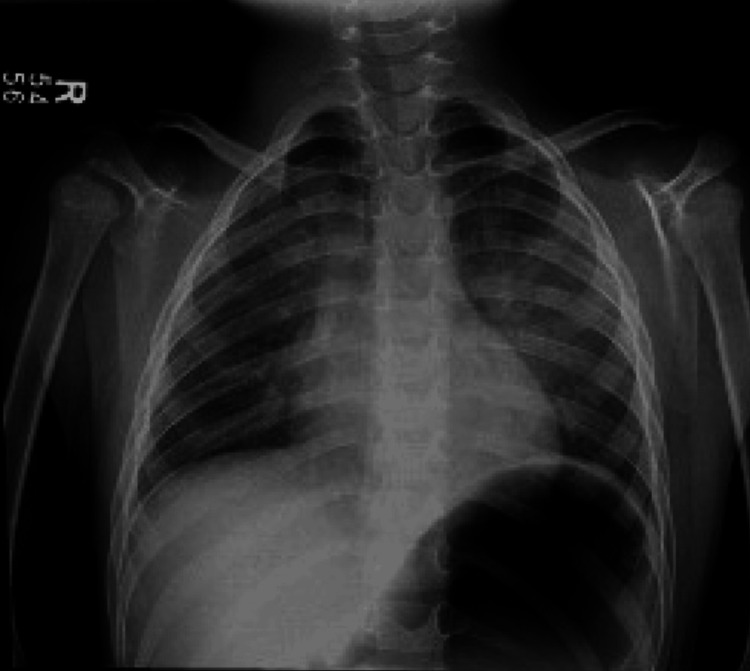
Postoperative chest X-ray showing clear lungs with no evidence of dental aspiration

**Figure 2:**
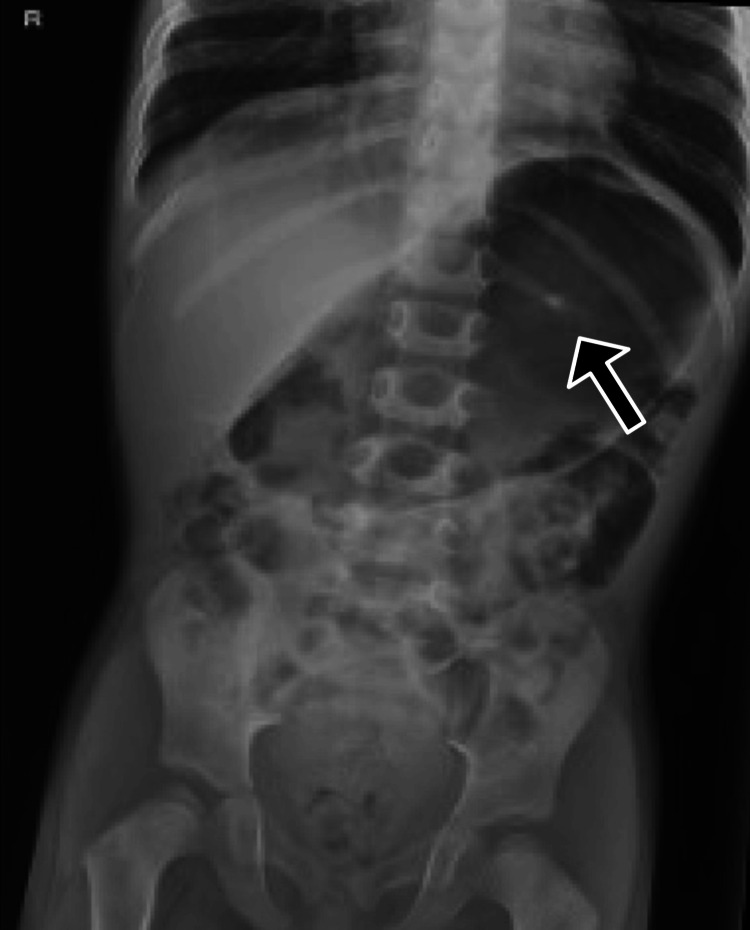
KUB radiograph with an arrow indicating the ingested tooth visualized in the stomach KUB: kidneys, ureters, and bladder

The surgery proceeded with no complications and was completed in 92 minutes. Suspension for airway exposure performed by the ENT surgeons proceeded without complications, providing optimal surgical visualization. The post-anesthesia evaluation was adequate, with the patient awake, alert, and airway patent after extubation. Shortly after arrival in the PACU, the grandmother of the child noted a missing central lower incisor. The anesthesiologist evaluated the patient and noted fresh gum in the area, confirming a recent loss of a tooth. A chest X-ray and a radiograph of the kidneys, ureters, and bladder (KUB) were ordered in the PACU due to concern about aspiration or ingestion of the tooth. The X-ray (Figure 1) showed no evidence of a foreign body in the airway or lungs. However, the KUB film (Figure 2) revealed a radiopaque object consistent with a tooth in the stomach. The ENT team and the family were promptly notified. The tooth was expected to pass naturally through regular bowel movements, and no further intervention was necessary. The family was reassured and instructed to monitor for any signs of GI distress and to follow up with their pediatrician.

## Discussion

This case underscores a critical and often underappreciated gap in standard anesthetic practice: the lack of routine postoperative dental inspection, particularly in pediatric patients. As described in the introduction, perioperative dental injuries are among the most frequent anesthesia-related adverse events and the leading cause of anesthesia malpractice claims. Despite their relatively low incidence, they carry disproportionate medico-legal and patient care implications. While most anesthesiologists are trained to assess for wiggly or cracked teeth preoperatively, especially in children with primary dentition, conducting a thorough oral inspection after extubation is not standard practice [10]. Incorporating a checklist to confirm with the surgeon any missing teeth or oropharyngeal abnormalities during mouth suspension may help prevent unrecognized dental injuries. This case highlights the consequences of that omission: a lower central incisor was avulsed during a routine tonsillectomy and was only noticed postoperatively by the patient’s family member, prompting delayed imaging and management.

In pediatric populations, the vulnerability is heightened. As literature shows, children undergoing general anesthesia with endotracheal intubation are at a significant risk of TDIs, particularly due to multiple patient and procedural factors. Difficult intubation has been identified as the most critical risk factor, increasing the likelihood of TDIs by 50 times, while multiple intubation attempts further raise the risk by 2.27 times [5]. Children with primary dentition are 3.6 times more susceptible to TDIs compared to those with mixed dentition, likely due to the greater mobility of primary teeth and their weaker structural integrity [5].

With a higher incidence of injury during pediatric cases, it is important to consider the prevalence and cause of these injuries. In a study on oral complications with endotracheal intubation of hospitalized children, maxillary incisors were the most commonly affected teeth, with concussion (60%), avulsion (20%), and subluxation (20%) being the most frequent hard tissue injuries [5]. Soft tissue injuries were more prevalent than hard tissue injuries, with the tongue (46.6%), upper lip (33.3%), and floor of the mouth (20.1%) being the most commonly affected areas [5]. The Macintosh laryngoscope blade, commonly used for intubation, exerts significant force on the maxillary incisors, increasing the risk of fractures, subluxation, and avulsion, particularly when multiple intubation attempts are necessary. To mitigate this risk, anesthesiologists should consider alternative intubation techniques, such as video laryngoscopy or fiberoptic intubation, which reduce direct contact with the teeth. Additionally, covering the upper and lower central incisors with gauze can provide a protective barrier and help minimize excessive force during intubation. During extubation, anesthesiologists should carefully inspect the oral cavity, avoid excessive manipulation of the teeth, and ensure smooth removal of the ETT to minimize the risk of dental trauma. Establishing such protocols will enhance patient safety and comfort and mitigate medico-legal risks associated with unrecognized dental trauma in pediatric anesthesia.

## Conclusions

The existing literature primarily focuses on preoperative precautions. Guidelines emphasize assessing dental stability, recording pre-existing dental damage, and informing patients of potential risks. To address the commonly overlooked issue of postoperative dental trauma, particularly in pediatric patients, a structured postoperative oral examination should be incorporated into routine anesthesia practice. Immediately following extubation and once the patient is stable, the anesthesiologist should conduct a focused inspection of the oral cavity, giving special attention to the upper and lower central incisors, as these are the most frequently affected by trauma during intubation. The evaluation should include a visual assessment for missing, displaced, or fractured teeth, as well as signs of recent trauma such as fresh gum exposure, bleeding, or swelling. If tolerated, gentle palpation may help assess the stability of any suspicious teeth, though care should be taken to avoid manipulating already loosened or avulsed teeth. Any findings should be clearly documented in the medical record, including comparisons to the preoperative dental assessment. If a tooth is found to be missing or suspected to have dislodged, immediate imaging, typically a chest X-ray and KUB radiograph, should be obtained to rule out aspiration or ingestion. For non-radiopaque dental materials or unclear cases, endoscopic evaluation may be required. Once the patient is sufficiently awake, the family should be informed of the findings promptly and compassionately, including an explanation of the injury, the steps taken to address it, and any recommendations for follow-up care. A dental consultation should be arranged prior to discharge, and the family should receive a written summary of the treatment plan, along with contact information for further care. Implementing this protocol would ensure early detection and appropriate management of perioperative dental injuries, improve communication with families, and help mitigate the legal and emotional consequences of undetected trauma.
